# Lamb Wave Line Sensing for Crack Detection in a Welded Stiffener

**DOI:** 10.3390/s140712871

**Published:** 2014-07-18

**Authors:** Yun-Kyu An, Jae Hong Kim, Hong Jae Yim

**Affiliations:** 1 International Institute for Urban Systems Engineering, Southeast University, Nanjing 210096, China; E-Mail: ayk2028@gmail.com; 2 School of Urban and Environmental Engineering, Ulsan National Institute of Science and Technology, Ulsan 689-798, Korea; E-Mail: jaekim@unist.ac.kr; 3 Department of Civil and Environmental Engineering, Korea Advanced Institute of Science and Technology, Daejeon 305-701, Korea

**Keywords:** nondestructive testing, structural health monitoring, Lamb wave crack detection, line sensing, welded stiffener, frequency-wavenumber analysis

## Abstract

This paper proposes a novel Lamb wave line sensing technique for crack detection in a welded stiffener. The proposed technique overcomes one of the biggest technical challenges of Lamb wave crack detection for real structure applications: crack-induced Lamb waves are often mixed with multiple reflections from complex waveguides. In particular, crack detection in a welded joint, one of the structural hot spots due to stress concentration, is accompanied by reflections from the welded joint as well as a crack. Extracting and highlighting crack-induced Lamb wave modes from Lamb wave responses measured at multi-spatial points along a single line can be accomplished through a frequency-wavenumber domain analysis. The advantages of the proposed technique enable us not only to enhance the crack detectability in the welded joint but also to minimize false alarms caused by environmental and operational variations by avoiding the direct comparison with the baseline data previously accumulated from the pristine condition of a target structure. The proposed technique is experimentally and numerically validated in vertically stiffened metallic structures, revealing that it successfully identifies and localizes subsurface cracks, regardless of the coexistence with the vertical stiffener.

## 1. Introduction

Lamb waves have been popularly used for crack detection because they are sensitive to the incipient damage and capable of propagating over a relatively long distance with little attenuation [1,2]. When Lamb waves propagating along a structure encounter a crack, they undergo reflection, refraction, transmission and mode conversions. These wave interactions allow characterizing the crack [3]. Based on the understanding of the Lamb wave interactions with the crack, a number of crack detection techniques have been developed. The conventional techniques have focused on crack-induced propagating Lamb waves measured by piezoelectric transducers (PZTs) attached on a target surface. Ihn *et al.* utilized Lamb waves scattered from a fatigue crack to characterize fatigue crack growth using a PZT network [4], and Lu *et al.* identified crack location by measuring Lamb waves scattered from the crack [5]. Kim *et al.* tried to extract the mode conversion signals caused by a crack using two pairs of collocated PZTs installed on both surfaces of a structure [6], and An *et al.* extracted the mode conversion signals to identify a crack using a specially designed dual-PZTs attached on a single surface of a structure [7].

However, the application of these conventional Lamb wave techniques is often limited to simple plate-like structures because crack-induced Lamb wave features measured from spatially limited target points are prone to piling up the other Lamb wave signals reflected from complex structural boundaries. Indeed, Lamb waves are sensitively changed by not only a crack but also additional structural features. Moreover, Lamb waves reflected from the crack and additional structural boundaries might be all mixed in the measured Lamb wave responses, thus making it difficult to identify and localize the crack. Although the cracks in complex structures can be identified by simply detecting the deviation of the current data from the baseline data obtained from the pristine condition of a structure, this simple pattern recognition is not practical. Since operational and environmental variations of the system can also cause signal changes, the simple pattern comparison can produce false alarms [8,9]. For real applications, these challenging issues should be tackled in advance, because many of real structures have complex boundary conditions and are exposed to environmental variations.

A few literatures have dealt with the technical challenging issues. Masserey *et al.* proposed a surface crack detection technique for a plate with multiple stiffeners [10], but the crack was apart from the stiffeners. In reality, a crack is most likely initiated from the welding-induced heat affected zone (HAZ) which is one of the representative stress concentration zones in structures with welded stiffeners [11]. Kim *et al.* tried to detect a crack at HAZ of the welded stiffener by extracting crack-induced mode conversion signals using two pairs of collocated PZTs [12]. More recently, An *et al.* developed a dual-PZT based mode conversion extraction technique for crack detection in HAZ and applied to *in-situ* bridge monitoring [13]. However, these trials still have a number of technical limitations in that they cannot localize cracks and are significantly affected by PZT installation conditions, making them less attractive for real structure monitoring.

In this study, a new Lamb wave line sensing technique based on a frequency-wavenumber (*f-k*) domain analysis is proposed so that crack-induced Lamb wave signals are more practically isolated from Lamb wave responses obtained at multi-spatial points along a single line even when the stiffener and crack coexist. The proposed technique is experimentally demonstrated using a steel plate with a welded vertical stiffener. A single PZT is utilized for Lamb wave generation, and the corresponding responses are measured at multi-spatial points along a single line using a scanning laser Doppler vibrometer (LDV). Furthermore, a finite element (FE) analysis is employed for more specifically verifying the proposed technique. The discussions associated with the technical limitations and path forward are also addressed in this paper.

This paper is organized as follows: first, the development of the Lamb wave line scanning technique with the experimental investigation is dealt with in Section 2. Subsequently, Section 3 presents the technical discussion with the FE analysis. Finally, this paper is concluded with an executive summary and brief discussion in Section 4.

## 2. Development of a Lamb Wave Line Sensing Technique

This section explains how Lamb wave line sensing is accomplished and how crack-induced Lamb wave features are extracted from the line sensed Lamb wave responses. The details of the experimental configuration and analysis procedure are as follows.

### 2.1. Description of Experimental Setup

**Figure 1.**
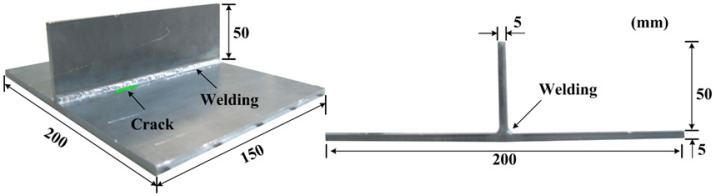
A vertically stiffened steel specimen with a crack.

First, a T-shape steel specimen with a welded joint is prepared to experimentally investigate the Lamb wave line sensing technique. The target specimen is fabricated by welding a vertical stiffener to a SS400 steel plate as shown in Figure 1. Using precision laser cutting, an artificial notch with a dimension of 10 × 0.25 × 0.99 mm^3^ is introduced at HAZ where the stress concentration is expected to occur [11]. Note that the artificial notch can properly represent a crack if the notch width is negligible compared to the smallest wavelength of the measured ultrasonic waves [14].

**Figure 2.**
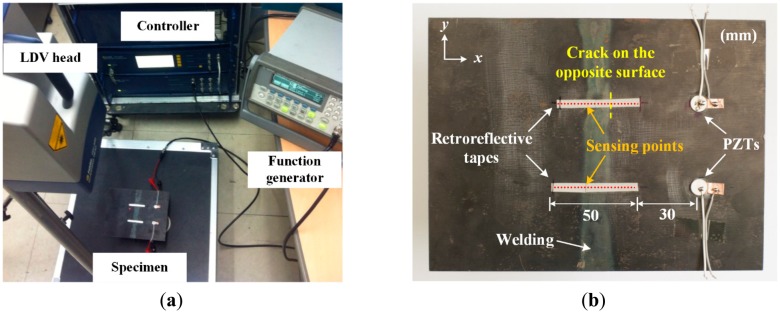
Experimental setup: (**a**) Overview and (**b**) Lamb wave generation and the line sensing schemes.

Figure 2a shows the overall experimental setup comprised of a function generator, LDV and controller. First, virtual measurement points on the target surface are created using a built-in digital camera of LDV, and the sensing sequences are predetermined. Then, the controller sends out a trigger signal to the function generator to excite PZT on the target specimen. The same trigger signal is simultaneously transmitted to LDV to activate data acquisition. Subsequently, the response signal is measured at a specific measurement point, transmitted to and stored in the control unit. Next, the control unit moves the sensing laser beam automatically to the next measurement point by sending control signals to the relevant galvanometer in LDV. By repeating the prescribed procedure, Lamb wavefields (*W_T_*) can be obtained at the target measurement points.

As for LDV, a commercial scanning LDV (PSV-400-M4, Polytec, Waldbronn, Germany) with a built-in galvanometer and an auto-focal lens is used in the test [15]. The laser source used for LDV is a He-Ne laser with a wavelength of 633 nm, and the minimum focal length of the auto-focal lens is 0.35 mm. The allowable scanning angle and scanning speed are ±20° and 2000°/s, respectively. This 1D LDV measures the out-of-plane velocity in the range of 0.01 μm/s to 10 m/s over a target surface based on the Doppler frequency-shift effect of light. Since the intensity of the signal laser beam reflected from the target surface highly depends on the surface condition, a special surface treatment is often necessary to improve the reflectivity of the returned laser beam.

A 7-cycle Hanning-windowed tone-burst input waveform with a driving frequency of 150 kHz generated by the function generator is used for Lamb wave excitation. The excitation voltage is 9 Vpp. Then, the corresponding responses are measured by LDV with a sampling rate of 5.12 MHz, and the sensitivity of the velocity measurement is set to 10 mm/s/V. The response signals are measured 30 times for each sensing point, averaged in the time domain, and bandpass-filtered with 10 kHz and 350 kHz cutoff frequencies to improve the signal-to-noise ratio. Here, retroreflective tapes are placed on the measurement points to enhance the reflectivity of the sensing laser beam. The distance from LDV to the target specimen is 900 mm.

The PZT installation and measurement scheme on the opposite surface to the vertical stiffener of the specimen are shown in Figure 2b. APC 850 type PZTs [16] with a diameter of 10 mm and a thickness of 0.508 mm are attached on the two different spatial points so that the test results obtained from intact and cracked areas can be effectively compared. The upper PZT and measurement points across the crack correspond to the crack case, while the lower setup represents the intact case on Figure 2b. Here, PZTs are 30 mm apart from the nearest measurement point. The spatial interval between the measurement points along each scanning line is 2 mm.

### 2.2. Frequency-Wavenumber (f-k) Domain Analysis

The *f-k* domain analysis has been recently used to analyze ultrasonic wavefields [17–21]. Its main advantage is that it can differentiate ultrasonic wavefields according to their propagation directions in a specific frequency range of interest. Thus, it is useful to analyze the wave scattering process caused by interacting with defects. This section explains how crack-induced Lamb wave features are extracted from *W_T_* measured at multiple spatial points where the welded vertical stiffener as well as a crack coexists. The main premise is that *W_T_* should be measured across the crack and stiffener locations. Once *W_T_* is collected from the target area, the *f-k* analysis is carried out as the following steps:

(1) Conversion of *W_T_* from the Time-Space (*t-s*) Domain to the *f-k* Domain
  The *f-k* domain analysis starts with the assumption that *W_T_* obtained along the 1D spatial domain (*x*-axis in this case) includes the target crack as well as stiffener. Then, *W_T_* is transformed from the *t-s* domain to the *f-k* domain using a 2D Fourier transform (FT):
  (1) $$w_{T}(k,\omega )=\int_{-\infty }^{\infty }\int_{-\infty }^{\infty }W_{T}(x,t)e^{-i(kx+\omega t)}\text{dxdt}$$
  where *w_T_* is Lamb wavefields in the *f-k* domain. *k*, *x*, ω and *t* denote wavenumber, spatial coordinate, angular frequency and time, respectively.
(2) Lamb Wave Filtering Depending on the Wave Propagation Direction in the *f-k* Domain
  To differentiate the wave components depending on the wave propagation direction, a tapered-cosine window function (Φ) is defined as:
  (2) $$\Phi (k,\omega )=\{\begin{array}{ll} 0 & \left|k(\omega )-k_{c}(\omega )\right|>2d(\omega )\\ 0.5+\text{0.5cos}\left[\frac{\pi \left\{k(\omega )-k_{c}(\omega )\right\}}{d(\omega )}\right] & \left|k(\omega )-k_{c}(\omega )\right|\leq 2d(\omega ) \end{array}$$
  where *k_c_* and 2*d* denote the center and width of Φ at a given ω.
  The selection of the center and width parameters of Φ is critical to properly isolate the crack-induced Lamb wave features. In this analysis, the Lamb waves reflected from the crack and propagating opposite to the incident waves are only considered. Because Lamb waves are physically more reflected from the waveguide-decreased crack formation than the waveguide-increased vertical stiffener, the crack-induced Lamb wave modes are able to be highlighted rather than the stiffener-induced ones by filtering only reflected Lamb wave modes. Once the *w_T_* values are projected on the *k* domain, the maximum and minimum *k* values covering the projected *w_T_* values are computed. Then, the *k_c_* and 2*d* are determined as the mean value between the maximum and minimum *k* values and the difference between the maximum and minimum *k* values, respectively. Then, the filtered wavefields (*w_f_*) in the *f-k* domain is computed as:
  (3) $$\begin{array}{cc} w_{f}(k,\omega )=w_{T}(k,\omega )\cdot \Phi (k,\omega ) & \forall \omega \end{array}$$
(3) Reconstruction of Lamb Wavefields in the *t-s* Domain
  The resultant Lamb wavefields (*W_f_*) in the *t-s* domain are reconstructed using an inverse 2D FT:
  (4) $$W_{f}(x,t)=\frac{1}{2\pi }\int_{-\infty }^{\infty }\int_{-\infty }^{\infty }w_{f}(k,\omega )e^{i(kx+\omega t)}\text{dkd}\omega$$
  The cumulative energy of *W_f_* is computed as:
  (5) $$E_{f}(x)=\int_{0}^{t}{\left[W_{f}(x,t)\right]}^{2}dt$$
  where *E_f_* represents the energy of *W_f_* accumulated up to a time point of *t*.
(4) Spatial Derivative and the Determination of Threshold Values
  Once *E_f_* is computed in the *s* domain, a spatial derivative is performed as:
  (6) Ef'(x)=∂Ef(x)∂x

The *E_f_* will be significantly altered if a crack exists along the wave propagation direction. Thus, *E_f_*′ abruptly increases where the crack is located. To highlight the crack formation, a threshold value is employed using an extreme value statistics [22]. First, the probability density function of *E_f_*′ is estimated by fitting a type I extreme value distribution known as a Gumbel distribution to all entities in *E_f_*′, and then the threshold value corresponding to a 99% confidence interval is computed. Subsequently, only the *E_f_*′ values above the computed threshold value are retained, making it possible to highlight the crack location and deemphasize undesired noise components.

### 2.3. Experimental Results

**Figure 3.**
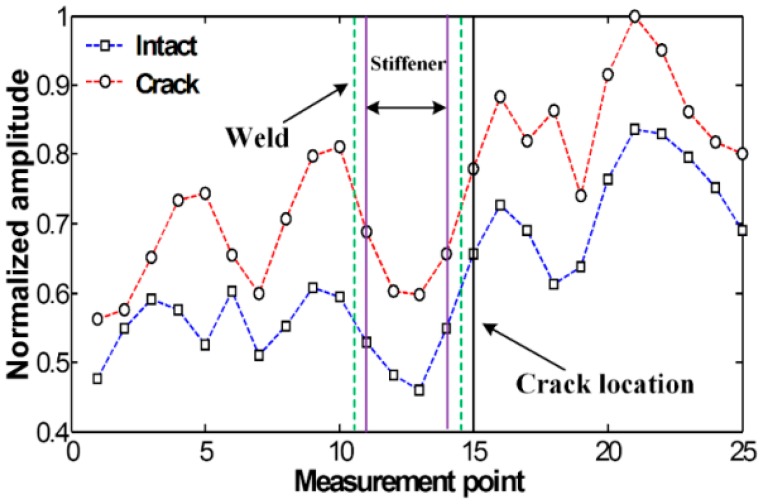
Spatial variations of cumulative Lamb wave energy obtained from the intact and cracked areas of the steel specimen.

Figure 3 compares the spatial variations of cumulative Lamb wave energies obtained between intact and cracked areas of the specimen. Experimental result shows that the intact and cracked energy distributions have similar patterns decreasing the node number as the measurement point moves further away from PZT.

No significant pattern difference is observed although amplitudes are different between intact and crack cases. The amplitude difference mainly comes from the variations of PZT bonding condition and LDV measurement errors. Small but existing experimental errors are caused by several noise sources such as PZT imperfection, LDV measurement noise and surface irregularity due to the welding.

**Figure 4.**
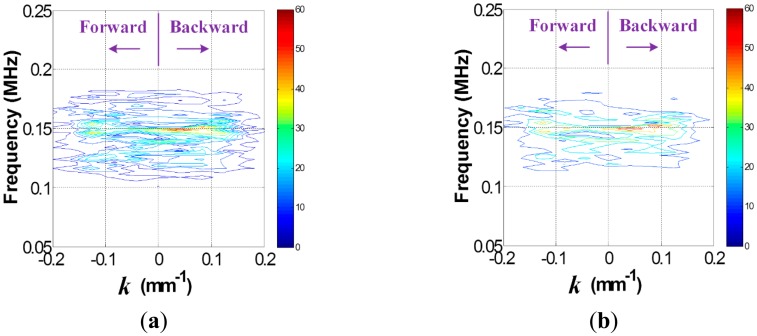
*f-k* domain plots obtained from the (**a**) intact and (**b**) cracked areas of the steel specimen.

**Figure 5.**
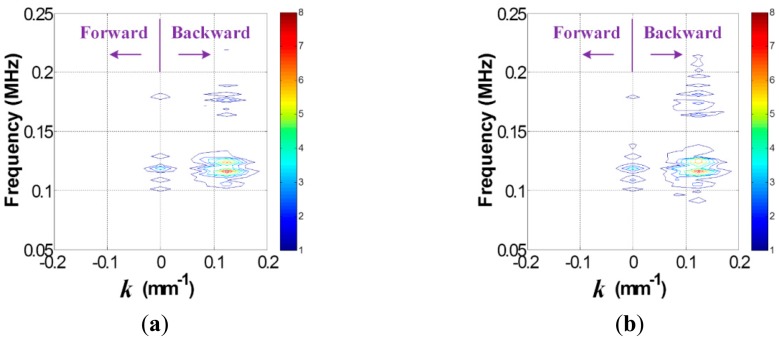
Filtered *f-k* domain plots obtained from the (**a**) intact and (**b**) cracked areas of the steel specimen.

Once the data in the *t-s* domain is converted to the *f-k* domain one using Equation (1), the *f-k* domain plots can be obtained as shown in Figure 4. Comparison of Figure 4a,b reveal that transmitted and reflected wave patterns, which are also represented by the forward and backward propagating waves in Figure 4, are different between intact and crack cases. Here, the forward and backward propagating directions are defined as −*x* and *x* directions in Figure 2b, respectively. To accentuate crack-reflected waves, only the backward propagating Lamb wave modes are filtered by applying Equations (2) and (3). Figure 5 shows the filtered *f-k* domain plots, highlighting crack-reflected wave modes.

**Figure 6.**
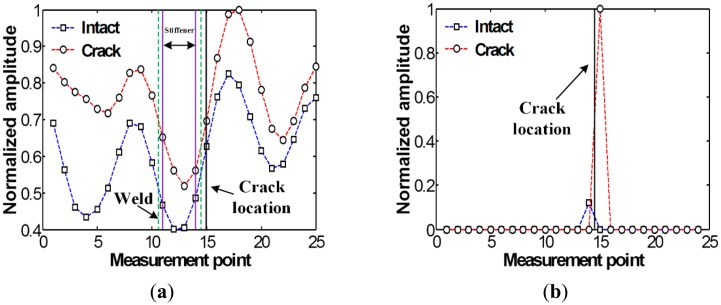
Spatial energy variations obtained from the intact and cracked areas of the specimen: (**a**) Filtered energy distributions and (**b**) the corresponding energy variations after spatial derivative and employing a threshold value.

Next, the filtered *f-k* domain data is reconverted to the *t-s* domain using Equation (4), and then their energy distribution computed using Equation (5) is displayed in Figure 6a. After applying the spatial derivative and thresholding processes described in the step 4 of Section 2.2 to Figure 6a, the crack location can be highlighted as shown in Figure 6b, indicating a good agreement with actual crack location. Here, the amplitudes are normalized using the maximum values of each graph. Note that although stiffener-induced energy concentration can also be observed nearby HAZ in the intact case, much smaller amplitude appear than the crack case.

## 3. Discussion

The proposed technique is also verified via a 2D FE analysis. In particular, the single line sensing of PZT-generated Lamb wave propagation in vertically stiffened plate models with and without a crack is simulated, and the Lamb wave interactions with a crack as well as a vertical stiffener are more thoroughly investigated. Through the FE analysis, technical strengths and limitations are also addressed.

### 3.1. Description of a 2D Finite Element (FE) model

**Figure 7.**
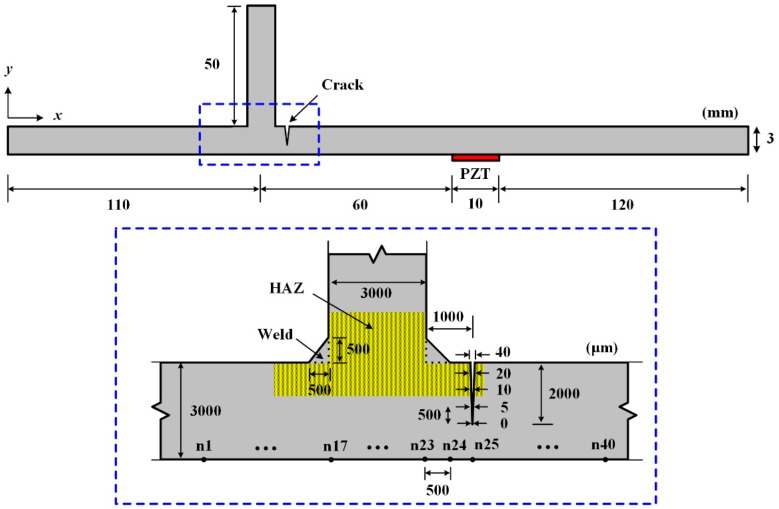
A 2D plane strain model with a vertical stiffener: PZT with a dimension of 10 × 0.508 mm^2^ is modeled on the target surface for Lamb wave generation, and a crack with a depth of 2 mm and widths varying from 0 to 40 μm is introduced at heat affected zone (HAZ). *n* indicates the inspection node number and 40 inspection nodes with an interval of 500 μm across the crack.

To validate the proposed technique, a 2D plane strain FE model with four-node bilinear quadrilateral (CPS4R) elements is made using ABAQUS/Standard 6.11 [23]. The 2D model demonstrates the vertically stiffened plate with an incipient crack, and an APC 850 type PZT [16] with dimensions of 10 × 0.508 mm^2^ is modeled on the opposite surface to the vertical stiffener and crack as shown in Figure 7. The material properties of the FE model are summarized in Table 1. The subsurface crack with a depth of 2 mm and widths varying from 0 to 40 μm along the through-the-thickness direction is introduced at HAZ as shown in Figure 7. Note that because the inspection surface is on the opposite side of the crack, the crack here is called a subsurface crack. The crack is modeled as a double-node [23]. The constraint conditions of the double-node between two crack interfaces are defined as follows: for normal behavior, the crack surfaces transmit contact stresses only when they are in contact, but no penetration is allowed at each constraint location. For tangential behavior, the relative sliding motion between the two crack surfaces is prevented as long as the corresponding normal contact constraints are active.

The PZT attached on the surface is used to generate Lamb waves by applying the input waveform of 7-cycle toneburst signals with the driving frequency of 300 kHz. The driving frequency is selected below A_1_ cutoff frequency. To guarantee proper simulation results, the spatial and time resolution should be well designed. The mesh size of 0.5 × 0.5 mm^2^ and the sampling rate of 20 MHz is determined by the spatial discretization rule [24]:

(7) $$\max(\Delta x,\Delta y)<\frac{\delta_{\min}}{10};\Delta t<\frac{0.7\min(\Delta x,\Delta y)}{C_{L}}$$

where Δ*x*, Δ*y* and δ_min_ represent *x*, *y* directional element dimensions and the shortest wavelength at a given frequency, respectively. Δ*t* denotes time interval.

To ensure the performance of the *f-k* domain analysis, inspection nodes should contain at least a single wavelength of Lamb wave mode. More than 36 discrete nodes with an identical spatial interval of 0.5 mm are required in this model because the wavelengths of fundamental symmetric (S_0_) and antisymmetric (A_0_) modes are about 17.60 mm and 10.56 mm, respectively. In this simulation, the out-of-plane displacements are measured at 40 discrete points across the crack and stiffened region as shown in Figure 7.

### 3.2. FE Simulation Results

**Figure 8.**
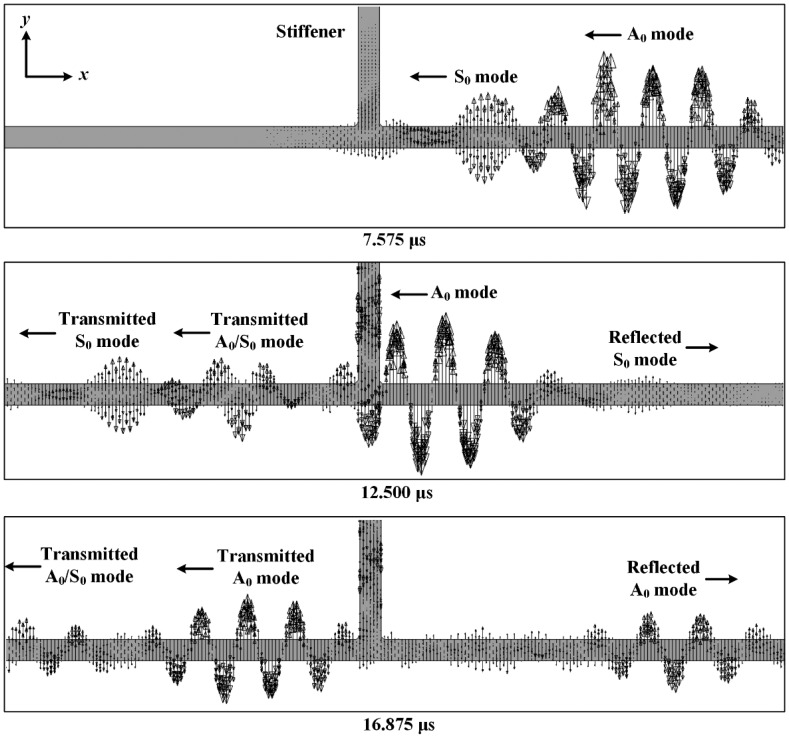
Representative Lamb wave interactions with a vertical stiffener.

First, Lamb wave interactions with a vertical stiffener are investigated using the simulation results obtained from the intact FE model. Figure 8 shows the representative out-of-plane response snapshots at different three time points. PZT generated Lamb waves propagating along −*x* direction starts to be separated into S_0_ and A_0_ modes at 7.575 μs due to their wave velocity difference.

When the incident S_0_ mode encounters the vertical stiffener at 12.500 μs, its propagation is separated into three different directions. First, a portion of the S_0_ mode is transmitted to and propagated along −*x* direction of the plate. Here, a part of the S_0_ mode is transmitted as the S_0_ mode, and the rest S_0_ mode is altered from S_0_ mode to A_0_ mode (denoted by A_0_/S_0_ mode) due to the stiffener, which is called mode conversion [25]. Second, another portion of the S_0_ mode is reflected from the vertical stiffener and propagates along *x* direction of the plate. During the reflection, the similar mode conversion phenomenon occurs although the converted modes are not clearly observed due to the dominant incident A_0_ mode. Third, the incident S_0_ mode propagates along the vertical stiffener. This leaked propagation behavior is also complicated, but the responses obtained at the stiffener is out of our interest in this study. Similarly, when the incident A_0_ mode passes by the vertical stiffener at 16.875 μs, a portion of the A_0_ mode is transmitted, reflected and leaked as the A_0_ mode or S_0_/A_0_ mode. These observations describe that the vertical stiffener acts as a considerable scatterer in the Lamb wave propagation.

**Figure 9.**
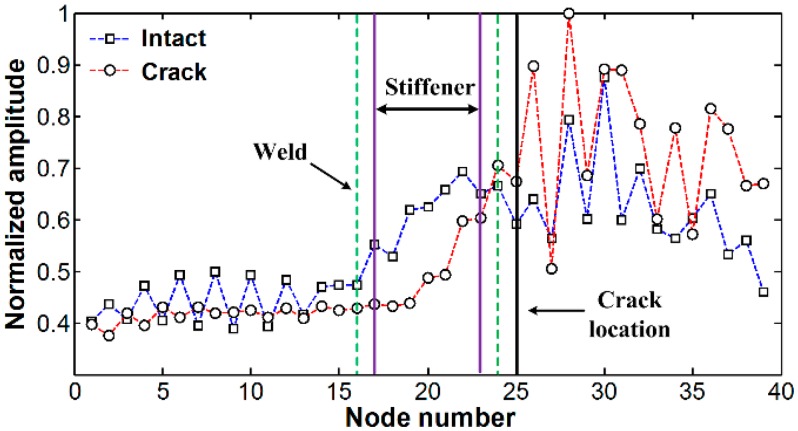
Spatial variations of cumulative Lamb wave energies obtained from the Finite Element (FE) models without and with the crack.

Figure 9 compares the spatial variations of cumulative Lamb wave energies computed using out-of-plane displacements obtained between the FE models without and with the crack. Note that out-of-plane displacements are only considered here so that the numerical results can be compared with the previous experimental results measured by 1D LDV which measures the out-of-plane displacements a target surface based on the Doppler frequency-shift effect of light. As expected, when Lamb waves are transmitted through the vertical stiffener, the sudden energy decreased near measurement points # 17 to # 23 is caused by leaking of Lamb waves along the stiffener. However, remarkable differences between the intact and crack cases cannot be unfortunately observed although more energy is concentrated in front of the crack location.

**Figure 10.**
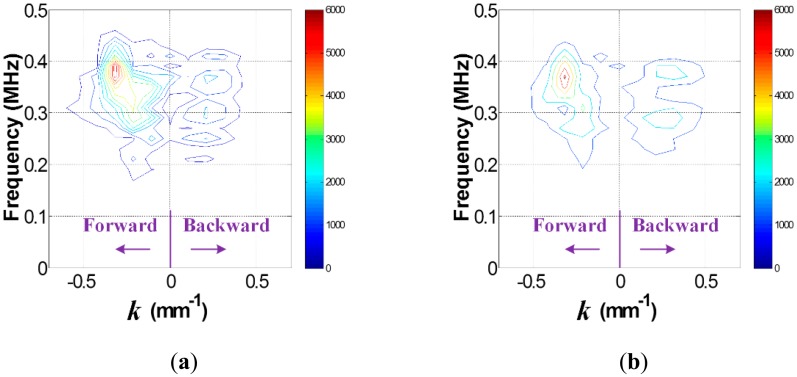
*f-k* domain plots obtained from the FE models (**a**) without and (**b**) with the crack.

**Figure 11.**
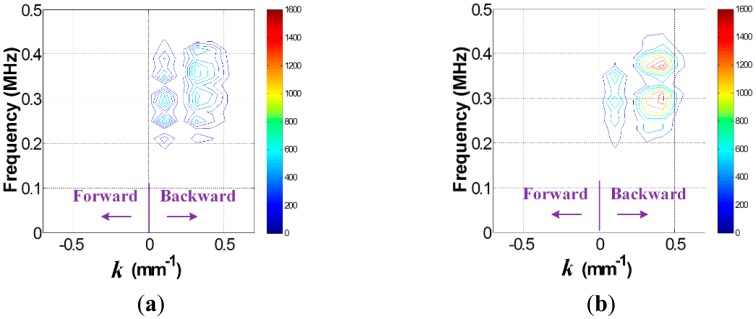
Filtered *f-k* domain plots obtained from the FE models (**a**) without and (**b**) with the crack.

Similarly, the *f-k* domain analysis is subsequently carried out. Figure 10 shows the *f-k* domain plots obtained using Equation (1). The forward and backward propagating Lamb wave modes appear differently. In particular, the backward propagating waves of Figure 11b have higher magnitude than that of Figure 11a after filtering, meaning that the crack case has much higher reflected energy than the intact case. Again, it can be physically understandable that additional Lamb wave modes created from the crack formation significantly contribute to the energy concentration only in the crack case.

**Figure 12.**
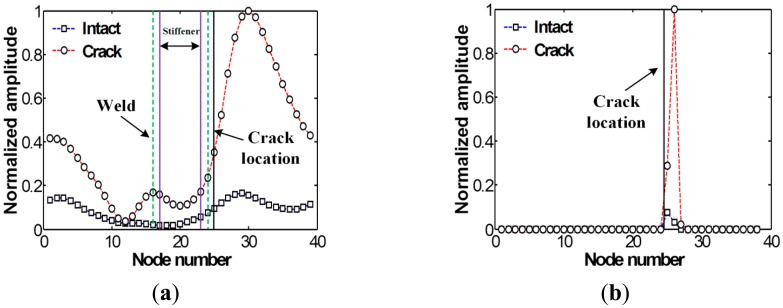
Spatial energy variations obtained from the FE models without and with the crack: (**a**) Filtered energy distributions and (**b**) the corresponding energy variations after spatial derivative and employing a threshold value.

Subsequently, the *t-s* domain plots reconverted from Figure 11 are shown in Figure 12a. Compared to Figure 9, crack-induced energy is more clearly highlighted in the crack case. Then, the crack location is highlighted after the spatial derivative and thresholding processes as shown in Figure 12b, showing a pretty good agreement with the previous experimental results. Note that the difference of the energy concentration pattern in the FE results is clearer than the test results as expected because there are no experimental errors in the simulation results.

Although the proposed technique offers promising crack identification and localization results, there are still some technical issues to be overcome for real-time monitoring of *in-situ* structures. First, crack identification and localization may not be accomplished without comparing between intact and cracked data because the amplitudes of Figures 6 and 12 are normalized with respect to their maximum values. Although crack-induced Lamb wave modes are successfully highlighted, positive false alarms can be indicated. This issue can be tackled by measuring the Lamb wave responses from several scanning lines. The responses measured at multiple scanning lines become baseline data to each other, making it possible to detect cracks using only currently measured data. To achieve this, a sophisticated statistical pattern recognition data processing algorithms, called baseline-free algorithms, are additionally necessary. This baseline-free crack diagnosis minimizes false damage alarms due to changing operational and environmental conditions by avoiding pattern comparisons with the baseline data previously obtained from the pristine condition of a target structure, which is now being developed.

## 4. Conclusions

This paper proposed a Lamb wave line sensing technique for crack detection in a welded stiffener. Its performance was numerically and experimentally validated by detecting subsurface cracks in vertically stiffened metallic structures. The proposed technique overcomes the technical limitations of the existing Lamb wave crack detection techniques for welded joint structures, contributing to the evolution of the Lamb wave nondestructive testing technique to real structural health monitoring applications. Although it has still some technical limitations addressed in the previous section, it is envisioned that automated and instantaneous crack alarms without user intervention can be subsequently accomplished based on the proposed technique. Further studies are warranted to address these issues.

## Figures and Tables

**Table 1.** Material properties of the plate model: Mass density (ρ), longitudinal wave velocity (*C_L_*), shear wave velocity (*C_T_*), Young's modulus (E), Poisson coefficient (υ) and the thickness of the plate (*t*).

**ρ (kg/m^3^)**	***C_L_* (m/s)**	***C_T_* (m/s)**	**E (GPa)**	**υ**	***t* (mm)**
2620.4	6370	3170	70	0.33	3
